# Clinical and Genetic Factors Associated with Resistance to Treatment in Patients with Schizophrenia: A Case-Control Study

**DOI:** 10.3390/ijms20194753

**Published:** 2019-09-25

**Authors:** Aline Hajj, Sahar Obeid, Saria Sahyoun, Chadia Haddad, Jocelyne Azar, Lydia Rabbaa Khabbaz, Souheil Hallit

**Affiliations:** 1 Laboratory of Pharmacology, Clinical Pharmacy and Drug Quality Control, Faculty of Pharmacy, Pôle Technologie-Santé (PTS), Faculty of Pharmacy, Saint-Joseph University, Beirut 1107 2180, Lebanon; lydia.khabbaz@usj.edu.lb; 2 Faculty of Pharmacy, Saint-Joseph University, Beirut 1107 2180, Lebanon; saria.sahyoun@gmail.com; 3 Faculty of Philosophy and Human Sciences, Holy Spirit University (USEK), Jounieh, Lebanon; Saharobeid23@hotmail.com; 4 Faculty of Pedagogy, Lebanese University, Beirut 14/6573, Lebanon; 5 Psychiatric Hospital of the Cross, Jal Eddib 6096, Lebanon; Chadia_9@hotmail.com (C.H.); Jossiek@hotmail.com (J.A.); 6 INSPECT-LB: Institut National de Sante Publique, Epidemiologie Clinique et Toxicologie, Beirut 1107 2180, Lebanon; souheilhallit@hotmail.com; 7 Faculty of Medicine, Lebanese American University, Byblos 13-5053, Lebanon; 8 Faculty of Medicine and Medical Sciences, Holy Spirit University of Kaslik (USEK), Jounieh, Lebanon

**Keywords:** BPRS, *COMT*, *DRD2*, MTHFR, OPRM1, PANSS, resistance, response to treatment, schizophrenia

## Abstract

Objectives: To assess clinical and genetic factors affecting response to treatment in a sample of patients with schizophrenia (treatment-resistant patients versus treatment responders). We also aimed at examining if these factors are different when we consider two different resistance classifications (the positive and negative syndrome scale, PANSS and the brief psychiatric rating scale, BPRS). Material and Methods: A case-control study included treatment-resistant patients and good responders. Patients were stratified in two groups based on the established criteria for treatment-resistant schizophrenia using BPRS and PANSS. The study was approved by the ethical committees (references: CEHDF1017; HPC-017-2017) and all patients/legal representatives gave their written consent. Clinical factors were assessed. DNA was obtained using a buccal swab and genotyping for *OPRM1*, *COMT*, *DRD2* et *MTHFR* genes using the Lightcycler^®^ (Roche). Results: Some discrepancies between the BPRS and PANSS definitions were noted in our study when assessing the patients’ psychopathological symptoms and response to treatment. The multivariable analysis, taking the presence versus absence of treatment resistance as the dependent variable, showed that that family history of schizophrenia, university studies, time since the beginning of treatment and chlorpromazine equivalent dose as well as the *COMT* gene are associated with resistance to treatment. In addition, a gender-related difference was noted for *COMT* SNP; men with at least one Met allele were more prone to be resistant to treatment than Val/Val patients. Conclusion: Uncovering the clinical and genetic factors associated with resistance to treatment could help us better treat our schizophrenic patients in a concept of personalized medicine.

## 1. Introduction

Schizophrenia (SCZ) is a severe neurodevelopmental disorder affecting 1% of the general population worldwide [1]. It represents a major concern of public health with very high rates of resistance to treatment. In fact, despite the introduction of atypical antipsychotics (especially clozapine) and despite all efforts implemented to individualize treatment, studies have shown that almost up to 30% of patients do no respond to treatment and still have a relapse during the first years of maintenance treatment.

Treatment-resistant schizophrenia is primarily defined by the severity of symptoms (positive) and the response to antipsychotics. A variety of representative scales, such as the positive and negative syndrome scale (PANSS), the brief psychiatric rating scale (BPRS), the scale for the assessment of negative symptoms (SANS) and the clinical global impression (CGI) clinical impression, were designed primarily to measure symptoms [1,2]. Using these different scales, studies have shown that treatment-resistant patients develop persistent positive psychotic symptoms, more pronounced negative symptoms and more severe cognitive impairment compared to treatment-responsive patients. Correctly identifying patients with resistance and understating the factors affecting the response to treatment could help health-care professionals better manage the patients’ disease thus reducing both health and economic burden to the patients, their families and the society [3,4]. In fact, studies have shown that individuals with treatment-resistant schizophrenia expressed the highest impairment in community functioning, low levels of achievements in functional milestones of everyday living, with high rates of unemployment, lack in psychological adjustment and sentimental relationship, leading to poorer quality of life and longer hospitalization rates/stays compared to non-resistant patients [4,5,6,7,8,9]. Heterogeneity of response to treatment is at the core of this challenge and different factors have been accounted for this variability including environmental and genetic factors [10]. Clinical and social factors include the age of onset of the disease, the duration of illness, the severity of the psychotic symptoms, the compliance to the pharmacological treatment and to non-pharmacological interventions [4,10,11,12].

Several candidate genes have been explored in pharmacogenomics studies of response to treatment in schizophrenia including genetic variants of the dopamine receptors and pathways/signaling genes. Hence, it is clearly stated nowadays that patients with schizophrenia have an altered dopaminergic function and several authors have shown that, in treatment-resistant patients, distinct dopamine changes could be further identified: lower density of dopaminergic synapses in the caudate nucleus, lower dopamine synthesis capacity in the striatum [13], and a decrease in the dopamine transporter protein expression, compared to patients who respond to antipsychotics. Moreover, numerous epidemiological and clinical studies suggested the role of inflammation in schizophrenia: authors speculated that pro-inflammatory cytokines may influence dopaminergic and glutaminergic pathways and cognition processes that are particularly altered in treatment-resistant patients [14,15,16,17,18,19].

Catechol-*O*-methyltransferase (COMT) is an enzyme that metabolizes catecholamines and is a key modulator of cortical dopaminergic degradation [20]. A common functional variant in the *COMT* gene have been studied in particular: the c.472G > A polymorphism (rs4680; p.Val158Met) causes a valine (Val) to methionine (Met) substitution at codon 158 in the membrane-bound isoform enzyme, leading to a three- to four-fold reduced activity of the enzyme [21,22], lower protein expression [21] and higher dopamine activity [23] for the Met variant compared to the Val variant. In a study examining the expression levels of *COMT* mRNA in post mortem cerebellum samples derived from psychiatric patients, including those with schizophrenia, the authors failed at identifying differences in *COMT* expression or methylation in any psychiatric disorder. However, a strong sexual dimorphism in its expression was identified and a reduced expression with some *COMT* SNPs such as rs737865 and rs165599 but not rs4680 [24]. A recent study evaluated gene expression of 13 genes including *COMT* in a context of resistance to treatment [25]: no differences could be noted between patients with or without treatment-resistance schizophrenia in whole blood gene expression. The relationship between *COMT* polymorphisms and response to antipsychotics have been extensively addressed in the literature, however, only a few studies addressed the *COMT* rs4680 in relation to resistance to treatment [6,26,27]. These studies yielded inconsistent results with relatively small sample sizes.

Another relevant gene that has been identified as an important modulator of response to treatment is the *DRD2* gene encoding the dopamine receptor 2, which is the most important target for antipsychotics. In acute schizophrenia patients, the mRNA expression levels of *DRD2* in peripheral blood samples were shown to be significantly lower than those in the healthy controls [28], but higher in chronic schizophrenia patients receiving long-term clozapine treatment [29].

Several studies have explored the association of single nucleotide polymorphisms (SNPs) in the genes encoding the dopamine receptors with the therapeutic effects of antipsychotics [7,30,31]. One particular study investigated the association between SNPs in *DRD2* (rs1801028 and rs179932) and resistance to treatment but no significant difference was noted [6]. We, therefore, thought to evaluate another SNP, the rs6277 that has never been explored in resistance to treatment.

Moreover, methylenete trahydrofolate reductase (MTHFR) is a pivotal enzyme that controls the intracellular methylation reactions, a key regulator to the production of neurotransmitters such as dopamine. It plays an essential role as well in the homocysteine level and neuroinflammation that could contribute to cognition impairment [32,33]. A single nucleotide polymorphism in *MTHFR* (SNP; c.677C > T; p.Ala222Val; rs1801133) reduces MTHFR activity and may influence dopamine signaling by exacerbating underlying cortical dopamine deficiency in schizophrenia patients [34]. The 677T variant has been associated with increased schizophrenia risk [35,36], more pronounced negative symptoms [37], and more severe executive dysfunction in these patients [37,38]. In addition, it has been extensively studied when exploring the side effects of antipsychotics in schizophrenic patients, in particular, the metabolic syndrome [39]. However, none of the studies have evaluated its association with resistance to treatment.

Finally, variants in the *OPRM1* gene encoding the μ-opioid receptor are of interest in the study of resistance to treatment as opioid receptors have been reported to regulate mesolimbic dopaminergic neuronal activities. Thus, the activation of μ-opioid receptors enhances extracellular dopamine concentration in the nucleus accumbens, which is known to be one of the main structures controlling physiological responses, behavior, and diseases including schizophrenia [40]. No previous studies have evaluated the role of the *OPRM1* c.118A > G SNP (rs1799971) in resistance to treatment in schizophrenic patients.

Since the definition for treatment-resistance has varied across different studies [10,41,42] and there is inconclusive or insufficient evidence of the association of all these SNPs in dopamine pathways genes and resistance to treatment and furthermore, none of the studies evaluated all these polymorphisms along with clinical factors, we conducted this case-control study to assess clinical and genetic factors affecting response to treatment in a sample of patients with schizophrenia (treatment-resistant patients versus treatment responders). We also aimed to examine if these factors are different when we consider two different resistance classifications (PANSS and BPRS).

## 2. Results

### 2.1. Demographic and Clinical Data of Patients

Among the 100 schizophrenic patients included in this study (73 women and 27 men; mean age of 49.35 ± 12.56 years), 53 met the criteria for treatment-resistant and 47 were treatment-responsive. Only, 4.2% (4) of the patients declared consuming psychoactive substances (cannabis, cocaine, hashish), 10.3% (10) alcohol, and 61.4% (62) were smokers. Regarding psychiatric disorders, 33.3% (31) of patients had a family history of schizophrenic episodes, and 29.5% (26) of other psychiatric conditions (dementia, personality disorders as paranoia, intellectual disability, depression, bipolar disorders and autism spectrum disorders). Almost all patients (88.4%; 84) had a personal history of schizophrenic episodes, whereas 18.1% (13) of them had a history of other psychiatric disorders (including bipolar disorder, anxiety, anorexia, and depression). Sociodemographic data of the patients are presented in Table 1. For the administered treatment, the mean treatment duration was 2.48 ± 1.89 years.

The most commonly used antipsychotic drugs were haloperidol (*n* = 91), promethazine (*n* = 52), chlorpromazine (*n* = 49), zuclopentixol (*n* = 37) and clozapine (*n* = 21). The mean calculated chlorpromazine-equivalent daily dose was 1210.83 ± 1247.74 mg for the whole sample.

### 2.2. Genotype and Allele Distribution

In order to determine the genetic background of the patients, genotype and allele frequencies of the studied SNPs were calculated and compared with other populations. Results are summarized in Table 2. The population was in Hardy-Weinberg equilibrium for the studied SNPs.

### 2.3. Variable Associated with Resistance to Treatment

Bivariate analyses were conducted to explore the variables associated with resistance to treatment taking each time the BPRS, the PANSS or both BPRS and PANSS criteria. Results are presented in Table 3, Table 4 and Table 5.

The bivariate analysis showed that resistance to treatment was significantly associated with male gender (48.9% vs. 7.5%), the consumption of psychoactive substances (9.3% vs. 0%), a higher mean of chlorpromazine equivalent daily dose (1466 vs. 984 mg), a lower mean adherence to treatment score (5.37 vs. 6.22) and a lower mean time since the beginning of treatment in years (1.85 vs. 2.84). Furthermore, when selecting males alone, the results showed that the *COMT* VM and MM genotypes were associated with resistance to treatment.

### 2.4. Gender Differences

A significantly higher percentage of men with the *COMT* VM genotype (52.2% vs. 0%) and the MM allele (26.1% vs. 0%) had resistance to treatment according to the BPRS scale (*p* = 0.008). The same results were found when resistance to treatment was assessed according to the PANSS scale (*COMT* VM allele: 56.3% vs. 27.3% and MM allele: 31.3% vs. 9.1%; *p* = 0.023). However, this association was not significant in women.

### 2.5. Multivariable Analyses

The results of the multivariable analyses are given in Table 6. A first logistic regression, taking the presence versus absence of treatment resistance according to the BPRS scale as the dependent variable, showed that a longer treatment (in years) (ORa = 0.730) was significantly associated with lower resistance to treatment (Table 6-model 1).

A second logistic regression, taking the presence versus absence of treatment resistance according to the PANSS scale as the dependent variable, showed that having a university level of education (ORa = 11.96), a positive family history of schizophrenia (ORa = 4.61) and a higher chlorpromazine equivalent dose (ORa = 1.001) were significantly associated with higher resistance to treatment (Table 6-model 2).

A third logistic regression, taking the presence versus absence of treatment resistance according to the PANSS or BPRS scales as the dependent variable, showed that a longer treatment (in years) (ORa = 0.67) was significantly associated with lower resistance to treatment, whereas a higher chlorpromazine equivalent daily dose (ORa = 1.001) was significantly associated with higher resistance to treatment (Table 6-model 3).

### 2.6. Gene-Gene Interaction

Several models were run to test for an association between the interactions of genes taken two by two and the resistance to treatment assessed by the BPRS, PANSS and BPRS or PANSS. No significant association was found between any of the interactions and the resistance to treatment (data not shown).

## 3. Methods

### 3.1. Study Design and Patients

This case-control study, conducted between October 2017 until February 2018, enrolled a convenient sample of 100 patients (resistant versus non-resistant) recruited randomly from the Psychiatric Hospital of the Cross (Lebanon). The schizophrenia in-patient database identified 300 in-patients as being eligible for inclusion in the study. After eligibility criteria were determined, subjects were assigned identification numbers and randomized according to an online software, Research Randomizer (www.randomizer.org). Patients aged more than 18 years, with a clinically confirmed diagnosis of schizophrenia (based on the Diagnostic and Statistical Manual 5th Edition DSM-5 criteria) were included. Schizophrenic patients with concomitant psychiatric disorders (depression and/or anxiety) were also included in the study and all details regarding their treatment were noted. Non-inclusion criteria consisted of refusal to enter the study, any pathology of the central nervous system affecting the cognitive function (multiple sclerosis, epilepsy, Parkinson’s disease, mental retardation, dementia, etc.), pregnancy and breastfeeding.

### 3.2. Compliance with Ethical Standards

The study was approved by the local ethical committees of Hotel-Dieu de France and the Psychiatric Hospital of the Cross hospitals (HDF-Reference: CEHDF1017 and HPC-reference: HPC-017-2017). All patients/legal representatives gave their written consent.

### 3.3. Clinical and Demographic Information

Clinical and demographic information included age, gender, ethnicity/nationality, marital status, education level, consumption of alcohol, tobacco and other psychoactive substances. All psychiatric details (related or not to schizophrenia) were noted: family/previous personal history of schizophrenic episodes, family/previous personal history of psychiatric disorders, time since the diagnosis of schizophrenia, number of episodes, start date of the actual episode, time since the start of antipsychotic treatment for the actual episode, anti-psychotic treatment (dose per 24 h), other co-medications, adherence to treatment, other non-pharmacological interventions (psychotherapy, social assistance, physical therapy, group therapy, ergotherapy, etc.). The chlorpromazine-equivalent daily dose of typical and atypical antipsychotics administered to patients was calculated according to published guidelines [52].

### 3.4. Response and Resistance to Treatment

Different scales were simultaneously used to assess the patients’ psychopathological symptoms, including the BPRS and the Arabic version [53] of the PANSS. The assessment was performed by a trained psychologist independent from the study. Patients were stratified in two groups based on the established criteria for treatment-resistant schizophrenia or treatment-responsive schizophrenia.

The BPRS is a widely used instrument allowing the assessment of the positive, negative, and affective symptoms of individuals with psychotic disorders, especially schizophrenia. It takes 20–30 min for the interview and scoring and consists of 18 symptom constructs. Answers are graded according to a Likert scale from 1 (not present) to 7 (extremely severe). Zero was entered if the item is not assessed. The BPRS has proven particularly valuable for documenting the treatment efficacy in patients who have moderate to severe disease. The BPRS scale was used as a primary end-point to assess resistance to treatment if the total BPRS score ≥45 for resistance with scores of at least four in two of the following items: conceptual disorganization, suspiciousness, hallucinatory behavior or unusual thought content, and a score of at least four (moderately ill) in the CGI scale.

We used the PANSS to measure symptoms’ severity in patients with schizophrenia and evaluate positive and negative symptoms of psychotic disorders. The scale includes 30 items, divided into 3 scales: 7 for the positive scale, 7 for the negative scale and 16 for the general psychopathological scale. The total score is calculated by summing the results for each question [54]. Resistance to treatment was considered when the PANSS score was ≥4 in at least two of categories: P2 (conceptual disorganization), P3 (hallucinatory behavior), P6 (suspiciousness/persecution), G9 (unusual thought content) (used as a secondary end-point).

### 3.5. Assessment of Adherence to Treatment

Adherence to treatment was assessed by asking the patients about the frequency, percent and rating response of their antipsychotics treatment use during the last month. Details are given in Supplementary Material 1. The total score was calculated by summing all three answers and presented as a percentage [55,56,57].

### 3.6. DNA Sampling and Genotyping

DNA was obtained using a buccal swab (Whatman^®^ FTA^®^ card technology-GE Healthcare) as recommended by the manufacturer. Genotyping of *OPRM1* (rs1799971), *COMT* (rs4680), MTHFR (rs1801133) and DRD2 (rs6275) was performed using the Lightcycler^®^ 2.0 (Roche Diagnostics GmbH-Mannheim-Germany). The PCR protocol and conditions are presented in Supplementary Table S1 and Table S2. Genotyping was performed in the laboratory following the patient’s inclusion and evaluation at the hospital. None of the investigators, clinical care providers or observers of this study were aware of the genotyping results, therefore, the genetic testing could not have biased the resistance assessment process.

### 3.7. Data and Statistical Analysis

All data were collected and processed by Statistical Package for the Social Sciences SPSS, Version 23. Categorical variables were presented as frequencies and percentages, and continuous variables as means with standard deviations. Statistical analysis was conducted using the Chi-square, Fisher exact t-test for dichotomous/categorical variables and the Student t-test for continuous variables. For the *COMT* gene, data were separately analyzed for males and females since sex-specific associations with *COMT* rs4680 have been previously reported [58,59]. In addition, three multivariable logistic regressions were conducted to eliminate potential confounders taking the resistance according to BPRS, resistance according to PANSS and resistance according to BPRS or PANSS as dependent variables respectively. In addition, variables that gave a *p*-value < 0.1 in the bivariate analysis were taken as independent variables. A *p*-value < 0.05 was considered significant [60].

## 4. Discussion

In schizophrenia, responses to antipsychotic treatment are complex and understanding the clinical and genetic affecting the variability in response is still a major challenge in psychiatry today. We conducted this study to evaluate clinical and genetic factors associated with resistance to treatment among a sample of Lebanese patients with schizophrenia. 

In our study, we used both BPRS and PANSS scales and compared the factors that were significantly associated with the resistance definition stated by each of these scales. Surprisingly, we found some discrepancies between the two definitions. This inconsistency has been addressed by the “Treatment response and resistance in psychosis (TRRIP)” working group [10] who agreed that there is a considerable variation in current approaches used to define resistance to treatment, which can contribute to failures to replicate findings. This factor alone could lead to inconsistent clinical management and treatment delay.

Our study showed that non-responder patients with schizophrenia had specific clinical features/patterns: they were more often men, had family history of schizophrenia and consumed more psychoactive substances than non-resistant patients, even if these factors did not remain significant in the multivariable analysis. These results are consistent with previously published studies [4]. Hence, some authors argued that the age of onset of the disease varies by sex and determines the response to treatment, with men developing the disease earlier being more resistant to treatment [61]. Moreover, some studies have identified a history of family psychosis as a predictor of treatment-resistant schizophrenia [62,63,64]. Finally, patients with resistance had higher rates of smoking, alcohol and substance abuse [3,4].

Regarding treatment characteristics, our results showed that resistant patients have been treated for a shorter duration of time (for the present episode), had lower treatment adherence scores and higher chlorpromazine-equivalent daily doses. This could be explained by the fact that patients who started their treatment later, have not had the time to stabilize it yet (by adjusting the therapy: finding adequate doses, substituting molecules, using clozapine or adjuvant treatments, etc.) to overcome resistance. Therefore, these patients could suffer from what is called a higher “duration of untreated psychosis” (DUP) because their current treatment duration is relatively recent. This high DUP is associated with a poor response to antipsychotic treatment according to the studies of Perkins et al. [11,65]. Regarding the problem of adherence to treatment, it is well recognized to be the single largest source of unrecognized errors in studies of treatment resistance because poorly adherent patients could present false-positive “pseudo-resistance” [10,11]. However, this is not applicable to our patients since they were all were treated with a minimum duration of 12 weeks and a daily chlorpromazine equivalent daily dose higher than 600 mg.

Our study was the first to evaluate the allelic and genotypic frequencies of the *DRD2* SNP in the Lebanese population. Allelic frequencies observed were similar to those described in the Japanese and Chinese populations but not the Caucasian population. This is not surprising because even if the current majority of the Lebanese population is considered to be Arabs, many ethnic communities have undergone mixing in the course of history [66]. For the other genes, allelic frequencies were similar to those previously reported in the Lebanese population.

Among all studied genetic factors, the *COMT* p.Val158Met was the only one found to be associated with resistance to treatment, specifically in men. Patients with a Met allele (VM and MM genotypes) were more likely to be resistant to antipsychotic treatment. These results are consistent with the conclusions highlighted by three previous studies including treatment-resistant versus non-resistant patients, which demonstrated a higher frequency of the Met/Met genotype in patients with TRS [26,27,67]. Sagud et al. [27] identified a link between the Met/Met genotype and TRS in a group of 55 resistant female patients versus 331 non-resistant ones. Moreover, Inada et al. [26] showed that patients with a Met/Met genotype had higher odds of being in the TRS group and had significantly received higher chlorpromazine equivalent doses compared to other genotypes. However, the authors did stratify their analysis according to gender. Finally, Escamilla et al. [67] identified that treatment responders presented a higher frequency of the Val allele in comparison with patients in an ultra-resistance group (sample of 218 Mexican patients). Other studies did not find any association between this SNP of *COMT* and TRS [6,25].

An explanation that can be put forward is that patients with Met/Met genotype for *COMT* have a higher dopamine stimulation in the prefrontal cortex due to their fourfold lower functional enzyme activity [13,21,22]. The brain tries to decrease the release of dopamine in the striatum, in order to protect the brain from excessive dopaminergic stimulation which could lead to severity of symptoms treatment-resistance [13,27]. The identified gender difference could be explained as well by the hypothesis that estrogens may affect the activity and functionality of COMT by influencing its gene expression [58,59].

For the other studied genetic factors, our study remains the first one to explore the role of *OPRM1* and *MTHFR* variants with the resistance to treatment. Regarding *OPRM1*, a study stipulated that the studied polymorphism can influence the myelination of axons especially in cortical neurons, which may play a role in the pathogenesis of schizophrenia [68]. Another hypothesis demonstrated the role of opioid receptors, in particular, the μ-opioid receptor, in mesolimbic dopaminergic neuronal activities, known to be disrupted in patients with schizophrenia [40]. MTHFR, as well, is widely recognized to be an important factor for the COMT metabolism of catecholamines, including cortical dopamine [34]. Thus, the studied SNP, by reducing this cortical dopamine, could affect not only the symptoms of patients [35,36,37,38] but also their response to treatment. We failed to identify such associations in our study. Further larger studies may be required to better explore these gene variants.

Finally, very few studies have evaluated the gene-gene interaction and its impact on resistance to treatment in patients with schizophrenia [69]. Rajagopal et al. [69] have explored the interaction between *COMT* rs4680 and *DRD4* 120-bp duplication and demonstrated statistically significant epistasis between these polymorphisms and clinical response to clozapine. To the best of our knowledge, our study is the first to explore the interaction of these four genes affecting the dopamine transduction in the brain and their possible impact with the response to the treatment. Even though our study yielded a negative result, there is a need to replicate those findings in a larger independent sample; future studies exploring the functional effects of genes and polymorphisms would allow a better understanding of the mechanism(s) underlying their interaction.

### Limitations and Strengths

Some limitations could be raised in our study. Due to the characteristics of the included population, the data lacks some highly important information regarding the pathology and its progress: date of the first episode, date of diagnosis, age of onset of treatment of the first schizophrenic episode, and most importantly the duration of treatment resistance, etc. Moreover, we acknowledge that the sample size was relatively small for genetic analyses and not gender-matched, which could explain some of the negative results due to a low statistical power; nevertheless, it remains big compared to the Lebanese population. Further multi-centered studies, including a larger sample of schizophrenic patients matched for gender, are required to confirm and generalize our results. Finally, the rating scales we used did not allow an evaluation of the cognitive symptom domain that could be altered in treatment-resistant patients [10]. However, our study was the first to compare resistance to treatment using two different validated scales (PANSS and BPRS) and we correlated the results of each of these scales to a maximum number of socio-demographic, clinical and genetic factors. Furthermore, our study is the first to evaluate different polymorphisms in different genes that could potentially affect cortical dopamine pathways and explore the gene-gene interaction.

## 5. Conclusions

Despite all efforts made in the assessment and management of schizophrenia, resistance to treatment remains a challenging issue for health-care professionals with a detrimental impact on the quality-of-life of patients and their families. Our results also confirmed the need to be extremely careful when interpreting resistance to antipsychotic treatment due to the wide range of available definitions. It highlights as well the impact of clinical and genetic factors on TRS.

Pharmacogenomics in treatment-resistant schizophrenic patients has been only partially implemented in clinical settings, with the main genes involved in the response to antipsychotic treatment not being fully elucidated. Such studies are crucial in upraising the concept of personalized treatment in complex diseases like schizophrenia. Future robust studies should be conducted to optimize drug treatment starting the first episode, in an attempt to reduce future resistance rates.

## Figures and Tables

**Table 1:** Demographic and clinical data of patients.

		Frequency (%) *
Gender	Male	27 (27.0%)
	Female	73 (73.0%)
Marital status	Married	18 (19.4%)
	Single	68 (73.1%)
	Divorced	7 (7.5%)
Level of education	Primary	42 (51.2%)
	Secondary	32 (39.0%)
	University	8 (9.8%)
Consumption of psychoactive substances	No	92 (95.8%)
	Yes	4 (4.2%)
Alcohol consumption	No	87 (89.7%)
	Yes	10 (10.3%)
Smoking	No	35 (36.1%)
	Yes	62 (63.9%)
Family history of schizophrenic episodes	No	62 (66.7%)
	Yes	31 (33.3%)
Family history of other psychiatric disorders	No	62 (70.5%)
	Yes	26 (29.5%)
Personal history of schizophrenic episodes	No	11 (11.6%)
	Yes	84 (88.4%)
Personal history of other psychiatric disorders	No	59 (81.9%)
	Yes	13 (18.1%)
		**Mean ± SD**
Age (years)		49.35 ± 12.56
Time since the beginning of treatment (Years)		2.48 ± 1.89
Chlorpromazine equivalent-dose (mg)		1210.83 ± 1247.74
Number of episodes		6.96 ± 5.43
Alternative medicine:		
Psychotherapy	15 (17%)	
Social assistance	6 (6.8%)	
Art therapy	12 (13.6%)	
Ergotherapy	7 (8%)	
Physical therapy	7 (8%)	

* Some numbers do not sum up to 100 due to missing data.

**Table 2:** Genotype and allele frequencies of *DRD2, OPRM1*, *COMT* and *MTHFR* variants in our population with a comparison with other previously published data.

Gene dbSNP	Genotype Frequencies ^1^			Allelic Frequencies		*p* ^2^
***DRD2* rs6275**	**CC**	**CT**	**TT**	**C**	**T**	
Schizophrenic patients *n* = 92 ^3^ (current study, schizophrenia)	16 (17.4)	47 (51.1)	29 (31.5)	0.43	0.57	-
HapMap European *n* = 113 [43]	48 (42.5)	55 (48.7)	10 (8.8)	0.67	0.33	<0.001 *
HapMap Japanese = 86 [43]	22 (25.6)	39 (45.4)	25 (29)	0.48	0.52	0.4
HapMap Chinese *n* = 43 [43]	10 (23.3)	27 (62.8)	6 (13.9)	0.55	0.45	0.09
HapMap Sub-Saharan African *n* = 113 [43]	14 (12.4)	39 (34.5)	60 (53.1)	0.30	0.70	0.008 *
***OPRM1* rs1799971**	**AA**	**AG**	**GG**	**A**	**G**	
Schizophrenic patients *n* = 100 (current study, schizophrenia)	77 (77.0)	23 (23.0)	0 (0)	0.89	0.12	-
Lebanese patients *n* = 84 [44]	67 (79.8)	17 (20.2)	0 (0)	0.90	0.10	0.9
Lebansee patients *n* = 96 [45]	76 (79.2)	18 (18.8)	2 (2.1)	0.89	0.11	0.28
***COMT* rs4680**	**Val/Val**	**Val/Met**	**Met/Met**	**Val**	**Met**	
Schizophrenic patients *n* = 100 (current study, schizophrenia)	34 (34.0)	41 (41.0)	25 (25.0)	0.55	0.46	-
Lebanese patients *n* = 84 [44]	22 (26.2)	42 (50)	20 (23.8)	0.51	0.49	0.42
Lebanese patients *n* = 96 [45]	23 (24)	48 (50)	25 (26)	0.49	0.51	0.27
***MTHFR* rs1801133**	**CC**	**CT**	**TT**	**C**	**T**	
Schizophrenic patients *n* = 100 (current study, schizophrenia)	42 (42.0)	42 (42.0)	16 (16.0)	0.63	0.37	-
Lebanese patients *n* = 589 [46]	290 (49.2)	234 (39.7)	65 (11.0)	0.69	0.31	0.24
Lebanese patients *n* = 233 [47]	105 (45.0)	101 (43.3)	27 (11.6)	0.67	0.33	0.54
Lebanese patients *n* = 205 [48]	134 (65.3)	63 (30.8)	8 (3.9)	0.81	0.19	<0.0001 *

^1^ Value represents the number of patients with percentage shown in parenthesis; ^2^ *p* values were obtained using a χ^2^ test between the number of patients of each genotype compared to our study [49,50,51]; **^3^** the numbers did not sum up to 100 because some genotyping could not be done successfully from buccal swabs probably due to xerostomia induced by antipsychotics; * Statistically significant result.

**Table 3:** Bivariate analysis taking the resistance to treatment, as evaluated by the BPRS scale, as the dependent variable.

		Resistance to Treatment (BPRS)		
		No	Yes	*p*-Value
		Frequency (%)	Frequency (%)	
Gender	Male	4 (7.5%)	23 (48.9%)	**<0.001**
	Female	49 (92.5%)	24 (51.1%)	
Consumption of psychoactive substances	No	53 (100.0%)	39 (90.7%)	**0.023**
	Yes	0 (0.0%)	4 (9.3%)	
*COMT*	VV	18 (34%)	16 (34%)	0.991
	VM	22 (41.5%)	19 (40.4%)	
	MM	13 (24.5%)	12 (25.5%)	
*COMT (male)*	VV	4 (100%)	5 (21.7%)	0.008
	VM	0 (0%)	12 (52.3%)	
	MM	0 (0%)	6 (26.1%)	
*COMT (female)*	VV	14 (28.6%)	11 (45.8%)	0.298
	VM	22 (44.9%)	7 (29.2%)	
	MM	13 (26.5%)	6 (25.0%)	
*DRD2*	CC	9 (18%)	7 (16.7%)	0.529
	CT	23 (46%)	24 (57.1%)	
	TT	18 (36%)	11 (26.2%)	
*MTHFR*	CC	24 (45.3%)	18 (38.3%)	0.390
	CT	19 (35.8%)	23 (48.9%)	
	TT	10 (18.9%)	6 (12.8%)	
*OPRM1*	AA	41 (77.4%)	36 (76.6%)	0.928
	AG	12 (22.6%)	11 (23.4%)	
	GG	0 (0.0%)	0 (0.0%)	
		**Mean ± SD**	**Mean ± SD**	***p*-value**
Adherence to treatment score (over 8)		6.22 ± 1.28	5.37 ± 1.59	**0.005**
Time since the beginning of treatment (years)		2.84 ± 1.89	1.85 ± 1.74	**0.029**
Chlorpromazine-equivalent dose (mg)		984.11 ± 1299.53	1466.48 ± 1231.12	**0.053**

BPRS: brief psychiatric rating scale; SD: Standard deviation; numbers in bold indicate significant associations between variables.

**Table 4:** Bivariate analysis taking the resistance to treatment, evaluated by the PANSS scale, as the dependent variable.

		Resistance to Treatment (PANSS)		
		No	Yes	*p*-Value
		Frequency (%)	Frequency (%)	
Gender	Male	11(16.9%)	16(45.7%)	**0.002**
	Female	54(83.1%)	19(54.3%)	
Level of education	Primary	29(53.7%)	13(46.4%)	**0.036**
	Secondary	23(42.6%)	9(32.1%)	
	University	2(3.7%)	6(21.4%)	
Consumption of psychoactive substances	No	65(100.0%)	27(87.1%)	**0.003**
	Yes	0(0.0%)	4(12.9%)	
Family history of schizophrenic episodes	No	47(73.4%)	15(51.7%)	**0.040**
	Yes	17(26.6%)	14(48.3%)	
Psychotherapy	No	53(88.3%)	20(71.4%)	**0.050**
	Yes	7(11.7%)	8(28.6%)	
Treatment by benzodiazepines	No	41(63.1%)	29(82.9%)	**0.040**
	Yes	24(36.9%)	6(17.1%)	
Treatment by anticholinergic drugs	No	23 (35.4%)	3 (8.6%)	**0.004**
	Yes	42 (64.6%)	32 (91.4%)	
*COMT*	VV	23 (35.4%)	11 (31.4%)	0.822
	VM	27 (41.5%)	14 (40.0%)	
	MM	15 (23.1%)	10 (28.6%)	
*COMT (male)*	VV	7 (63.6%)	2 (12.5%)	0.023
	VM	3 (27.3%)	9 (56.3%)	
	MM	1 (9.1%)	5 (31.3%)	
*COMT (female)*	VV	16 (29.6%)	9 (47.4%)	0.273
	VM	24 (44.4%)	5 (26.3%)	
	MM	14 (25.9%)	5 (26.3%)	
*DRD2*	CC	10 (16.1%)	6 (20.0%)	0.897
	CT	32 (51.6%)	15 (50.0%)	
	TT	20 (32.3%)	9 (30.0%)	
*MTHFR*	CC	28 (43.1%)	14 (40.0%)	0.520
	CT	25 (38.5%)	17 (48.6%)	
	TT	12 (18.5%)	4 (11.4%)	
*OPRM1*	AA	52 (80.0%)	25 (71.4%)	0.331
	AG	13 (20.0%)	10 (28.6%)	
	GG	0 (0.0%)	0 (0.0%)		
		**Mean ± SD**	**Mean ± SD**	
Time since the beginning of treatment (years)		2.71 ± 1.92	1.85 ± 1.69	0.080
Chlorpromazine equivalent dose (mg)		1006.28 ± 1232.53	1590.71 ± 1202.11	**0.025**
Adherence to treatment score (over 8)		6.10 ± 1.37	5.31 ± 1.61	**0.010**

PANSS: positive and negative syndrome scale; SD: Standard deviation; numbers in bold indicate significant associations between variables.

**Table 5:** Bivariate analysis taking the resistance to treatment, evaluated by the BPRS or PANSS scales, as the dependent variable.

		Resistance to Treatment (BPRS or PANSS)		
		No	Yes	*p*-Value
		Frequency (%)	Frequency (%)	
Gender	Male	2 (4.0%)	23 (47.9%)	**<0.001**
	Female	48 (96.0%)	25 (52.1%)	
Consumption of psychoactive substances	No	50 (100.0%)	40 (90.9%)	**0.029**
	Yes	0 (0.0%)	4 (9.1%)	
Family history of schizophrenic episodes	No	38 (76.0%)	22 (53.7%)	**0.025**
	Yes	12 (24.0%)	19 (46.3%)	
*COMT*	VV	16 (32.0%)	16 (33.3%)	0.896
	VM	22 (44.0%)	19 (39.6%)	
	MM	12 (24.0%)	13 (27.1%)	
*DRD2*	CC	7 (14.9%)	8 (18.6%)	0.549
	CT	23 (48.9%)	24 (55.8%)	
	TT	17 (36.2%)	11 (25.6%)	
*MTHFR*	CC	22 (44.0%)	18 (37.5%)	0.609
	CT	19 (38.0%)	23 (47.9%)	
	TT	9 (18.0%)	7 (14.6%)	
*OPRM1*	AA	39 (78.0%)	37 (77.1%)	0.913
	AG	11 (22.0%)	11 (22.9%)	
	GG	0 (0.0%)	0 (0.0%)	
		**Mean ± SD**	**Mean ± SD**	
Adherence to treatment score (over 8)		6.40 ± 1.02	5.37 ± 1.58	**<0.001**
Time since the beginning of treatment (years)		2.85 ± 1.91	1.82 ± 1.72	**0.022**
Chlorpromazine equivalent dose (mg)		851.17 ± 821.37	1479.69 ± 1221.38	**0.004**

BPRS: brief psychiatric rating scale; PANSS: positive and negative syndrome scale; SD: Standard deviation; numbers in bold indicate significant associations between variables.

**Table 6:** Multivariable analyses.

**Logistic Regression 1: Taking Resistance to Treatment Based on the BPRS as the Dependent Variable**				
**Variable**	***p*-Value**	**ORa**	**95% Confidence Interval (CI)**	
			**Lower Bound**	**Lower Bound**
Time since the beginning of treatment (years)	**0.047**	0.730	0.535	0.996
Adherence to treatment score	0.094	0.679	0.432	1.068
Chlorpromazine equivalent daily dose (mg)	0.062	1.001	1.000	1.001
Variables entered in model 1: Gender, time since the beginning of treatment (years), adherence to treatment score, consumption of psychoactive substances, chlorpromazine equivalent daily dose (mg).				
**Logistic Regression 2: Taking Resistance to Treatment Based on the PANSS as the Dependent Variable**				
**Variable**	***p*-Value**	**ORa**	**95% Confidence Interval (CI)**	
			**Lower Bound**	**Lower Bound**
Level of education: Secondary	0.369	0.481	0.097	2.377
Level of education: University	**0.042**	11.962	1.095	130.661
Family history of schizophrenic episodes	**0.043**	4.617	1.048	20.329
Time since the beginning of treatment (years)	0.097	0.675	0.425	1.074
Chlorpromazine equivalent daily dose (mg)	**0.020**	1.001	1.000	1.002
Variables entered in model 2: Gender, level of education, family history of schizophrenic episodes, psychotherapy, time since the beginning of treatment (years), adherence to treatment score, consumption of benzodiazepines, chlorpromazine equivalent daily dose (mg).				
**Logistic Regression 3: Taking Resistance to Treatment Based on the BPRS or PANSS as the Dependent Variable**				
**Variable**	***p*-Value**	**ORa**	**95% Confidence Interval (CI)**	
			**Lower Bound**	**Lower Bound**
Time since the beginning of treatment (years)	**0.023**	0.675	0.481	0.946
Adherence to treatment score	0.068	0.646	0.405	1.032
Chlorpromazine equivalent daily dose (mg)	**0.026**	1.001	1.000	1.002
Family history of schizophrenic episodes	0.088	2.779	0.858	9.003
Variables entered in model 3: Gender, family history of schizophrenic episodes, time since the beginning of treatment (years), adherence to treatment score, consumption of psychoactive substances, chlorpromazine equivalent daily dose (mg).				

Numbers in bold indicate significant associations between variables; BPRS: brief psychiatric rating scale; PANSS: positive and negative syndrome scale; ORa = adjusted odds ratio; CI = confidence interval.

## Supplementary Materials

The following are available online at https://www.mdpi.com/1422-0067/20/19/4753/s1.
