# Supplementary material for: Clinical and Genetic Factors Associated with Resistance to Treatment in Patients with Schizophrenia: A Case-Control Study
Source: Int J Mol Sci. 2019 Sep 25;20(19):4753. doi: 10.3390/ijms20194753 (PMC6801865; doi:10.3390/ijms20194753)
Supplement: Supplementary file 1 [file ijms-20-04753-s001.pdf]

Supplementary material

Supplementary material 1: Assessment of adherence to treatment

Concerning the frequency, we asked the patient “did you take all your medications all the time?” with the possible responses being divided as follow: 0% for none of the time, 20% for a little of the time, 40% for some of the time, 60% for a good bit of the time, 80% for most of the time and 100% for all the time. The percent item was checked using the question “what percent of the time were you able to take your medications exactly as your doctor prescribed them?”. The rating item was assessed using the following question “rate your ability to take all your medications as prescribed” with the possible answers being divided as follows: 0% = very poor, 20% = poor, 40% = fair, 60% = good, 80% = very good and 100% = excellent.

Supplementary Table S1: The PCR protocol and conditions for *DRD2*, *OPRM1*, *COMT* and *MTHFR* genotyping using Lightcycler® 2.0 instrument.

The reaction was carried out using 25 ng of DNA (10 ng/µL solution or 2.5 µL) in a final volume of 10 µL. The reaction mixture (10 µL) contained Fast Start Taq polymerase (10×), buffer and dNTPs, $MgCl_2$ (10 mM); Lightcycler Fast Start DNA Master Hybridization Probes Kit® catalogue no. 03 003 248 001, Roche Diagnostics GmbH, Mannheim, Germany), and 0.5 µL of a Mix including both primers and fluorescent probes (anchor and sensor; TIB Molbiol®, TIBMOLBIOL, Berlin, Germany). The samples were then loaded into composite plastic/glass capillaries (20 µL LC capillaries, Roche Diagnosis, Roche Diagnostics GmbH, Mannheim, Germany), centrifuged, and placed in the LightCycler sample carousel.

Positive heterozygous and homozygous controls (defined by direct sequencing) and negative controls (water) were systematically included in all experiments.

	**PCR**				**FUSION CURVES**	
***DRD2* rs6277**	10 s at 95˚C	10 s at 60˚C	15 s at 72˚C	45 cycles	Decrease from 95 ˚C to 40˚C	Increase from 40˚C to 85˚C with a ramp of 0.2˚C/s
***OPRM1* rs1799971**	10 s at 95˚C	10 s at 60˚C	15 s at 72˚C	45 cycles	Decrease from 95 ˚C to 40˚C	Increase from 40˚C to 85˚C with a ramp of 0.2˚C/s
***COMT* rs4680**	10 s at 95˚C	10 s at 60˚C	15 s at 72˚C	45 cycles	Decrease from 95 ˚C to 40˚C	Increase from 40˚C to 85˚C with a ramp of 0.2˚C/s
***MTHFR* rs1801133**	5 s at 95˚C	10 s at 60˚C	15 s at 72˚C	45 cycles	Decrease from 95 ˚C to 43˚C	Increase from 43˚C to 75˚C with a ramp of 0.2˚C/s

<u>Supplementary Table S2:</u> Bivariate analysis taking the resistance to treatment, evaluated by the BPRS or PANSS, as a dependent variable.

		**Resistance to treatment (BPRS or PANSS)**		
		**No**	**Yes**	p-value
		**Frequency (%)**	**Frequency (%)**	
**Gender**	Male	2 (4.0%)	23 (47.9%)	**<0.001**
	Female	48 (96.0%)	25 (52.1%)	
**Consumption of psychoactive substances**	No	50 (100.0%)	40 (90.9%)	**0.029**
	Yes	0 (0.0%)	4 (9.1%)	
**Family history of schizophrenic episodes**	No	38 (76.0%)	22 (53.7%)	**0.025**
	Yes	12 (24.0%)	19 (46.3%)	
COMT	VV	16 (32.0%)	16 (33.3%)	0.896
	VM	22 (44.0%)	19 (39.6%)	
	MM	12 (24.0%)	13 (27.1%)	
DRD2	CC	7 (14.9%)	8 (18.6%)	0.549
	CT	23 (48.9%)	24 (55.8%)	
	TT	17 (36.2%)	11 (25.6%)	
MTHFR	CC	22 (44.0%)	18 (37.5%)	0.609
	CT	19 (38.0%)	23 (47.9%)	
	TT	9 (18.0%)	7 (14.6%)	
OPRM1	AA	39 (78.0%)	37 (77.1%)	0.913
	AG	11 (22.0%)	11 (22.9%)	
	GG	0 (0.0%)	0 (0.0%)	
		**Mean ± SD**	**Mean ± SD**	
**Adherence to treatment score (over 8)**		6.40 ± 1.02	5.37 ± 1.58	**<0.001**
**Time since the beginning of treatment (Years)**		2.85 ± 1.91	1.82 ± 1.72	**0.022**
**Chlorpromazine equivalent dose (mg)**		851.17 ± 821.37	1479.69 ± 1221.38	**0.004**

SD: Standard deviation

The numbers in bold represent the explanatory variables that showed associations to the resistance to treatment as evaluated by either BPRS or PANSS with $p < 0.1$ in the univariate analyses [46].
